# ^1^H NMR-based small molecular metabolites characterization and free fatty acid composition of Xuanwei ham and Jinhua ham

**DOI:** 10.1016/j.fochx.2025.103071

**Published:** 2025-09-23

**Authors:** Ruwei Ren, Ling Li, Guiying Wang, Jia Liu, Nannan Zhou, Wen Xun, Yanfei Du, Shuai Tang, Jiayan Tan, Guozhou Liao

**Affiliations:** a College of Food Science and Technology, Yunnan Agricultural University, Kunming 650201, China; b Livestock Product Processing and Engineering Technology Research Center of Yunnan Province, Yunnan Agricultural University, Kunming 650201, China

**Keywords:** Xuanwei ham, Jinhua ham, Free fatty acid, Small molecule metabolites, NMR

## Abstract

Xuanwei ham (XW) and Jinhua ham (JH), two representative Chinese dry-cured hams, exhibit distinct flavor profiles. This study combined ^1^H NMR metabolomics and GC–MS to compare their free fatty acid (FFA) compositions and small-molecule metabolites. XW showed higher retention of polyunsaturated fatty acids (PUFAs) and significantly more linoleic acid in subcutaneous fat (+39.29 %, *p* < 0.05), along with greater oxidative stability. In contrast, JH displayed severe lipid oxidation under high-temperature fermentation, as indicated by a sharp drop in intramuscular PUFA/SFA ratio. NMR revealed higher levels of umami-enhancing metabolites (taurine, glutamate, lysine) in XW. Multivariate analysis confirmed significant metabolic divergence between the hams, highlighting how raw materials and processing conditions shape their unique flavors.

## Highlights

• Combined ¹H NMR and GC-MS to compare Xuanwei and Jinhua ham metabolic and lipid profiles.
• Xuanwei ham showed superior oxidative stability and higher polyunsaturated fatty acid retention.
• Jinhua ham's high-temperature fermentation drove intense lipid oxidation and reduced PUFA/SFA ratio.
• Revealed how raw materials and processing conditions mechanistically shape distinct flavor profiles.

## Graphical abstract

**Figure:**
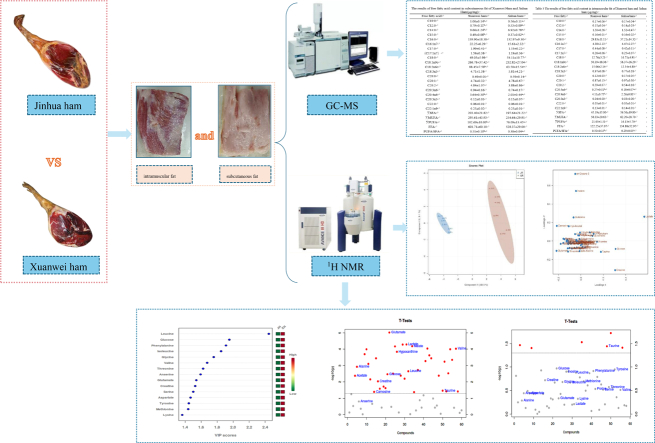

## 1 Introduction

Dry-cured ham is a traditional and highly valued meat product in China, with Xuanwei ham and Jinhua ham being two of the most renowned varieties. Xuanwei ham, originating from Yunnan Province, is celebrated for its light and delicate flavor, while Jinhua ham, from Zhejiang Province, is known for its strong and robust aroma. The distinct flavor profiles of these hams are influenced by a combination of factors, including pig breed, feed composition, curing methods, fermentation conditions, and aging processes (Li, Zhao, et al., 2024). Xuanwei ham is typically produced from hybrid pigs with Wujin pig lineage, whereas Jinhua ham is made from Jinhua pigs, a local breed known for its high intramuscular fat content and superior meat quality (Zhang et al., 2023). Despite their popularity, the underlying biochemical mechanisms responsible for their unique flavor characteristics remain poorly understood. Previous studies have provided foundational insights into the flavor profiles of Chinese dry-cured hams. For instance, Deng et al. (2022) delivered a comprehensive analysis of the correlation between characteristic flavor and microbial community in Jinhua ham during its post-ripening stage, highlighting the crucial role of microorganisms in flavor development. Similarly, Liu et al. (2019) conducted a pioneering ^1^H NMR-based metabolomic study, comparing the metabolic profiles of five kinds of Yunnan dry-cured hams, which advanced our understanding of regional differences.

However, a direct, systematic, and simultaneous comparison of the metabolic basis between Jinhua ham and Xuanwei ham the two most iconic representatives is still lacking. Specifically, the existing literature has not yet:Integrated the analysis of both the FFA composition and the water-soluble small-molecule metabolome to provide a holistic view of the flavor precursor pool.Investigated the potential correlations between the differential oxidation rates of unsaturated fatty acids (e.g., the significantly higher subcutaneous PUFA content in Xuanwei ham) and the divergent abundances of key taste-active metabolites (e.g., amino acids, organic acids) in these two hams.Elucidated how the distinct raw material (breed-specific traits) and processing techniques (e.g., Jinhua's high-temperature fermentation vs. Xuanwei's low-temperature maturation) interact to shape the final flavor profile through these biochemical pathways.This knowledge gap impedes a mechanistic understanding of what fundamentally dictates their distinct sensory characteristics (‘strong and robust’ vs. ‘light and delicate’).

Flavor development in dry-cured ham is a complex process driven by the degradation of proteins, lipids, and carbohydrates, as well as the subsequent formation of volatile and non-volatile compounds (Fu et al., 2022; Xiao, Ge, et al., 2019). Among these, small-molecule metabolites and FFA play a pivotal role in shaping the sensory attributes of ham. FFA, derived from lipid hydrolysis, serve as precursors for the formation of volatile flavor compounds through oxidation and other chemical reactions (Wu et al., 2017). Similarly, small-molecule metabolites, such as amino acids, organic acids, and nucleotides, contribute to the taste and aroma of ham by participating in Maillard reactions and Strecker degradation (Tian et al., 2022). Therefore, a comprehensive analysis of these components is essential for understanding the flavor differences between Xuanwei and Jinhua hams.

In recent years, metabolomics has emerged as a powerful tool for studying the chemical composition of food products. Nuclear magnetic resonance (NMR) spectroscopy, in particular, has been widely used in meat science due to its ability to provide detailed information on a wide range of metabolites in a non-destructive manner (Qi et al., 2023; Xiao, Luo, et al., 2019). When combined with multivariate statistical methods such as principal component analysis (PCA) and orthogonal projections to latent structures discriminant analysis (OPLS-DA), NMR-based metabolomics can effectively identify and quantify metabolic differences between samples (Ahmed et al., 2022). This approach has been successfully applied to analyze the metabolic profiles of various dry-cured hams, providing insights into the biochemical processes underlying flavor formation (Bajd et al., 2016; Zhou et al., 2021).

Despite the growing body of research on dry-cured hams, there is a lack of systematic studies comparing the metabolic profiles of Xuanwei and Jinhua hams. Such comparisons are crucial for elucidating the factors responsible for their distinct flavor characteristics. Therefore, this study aims to investigate the differences in FFA composition and small-molecule metabolites between Xuanwei and Jinhua hams using ^1^H NMR spectroscopy and gas chromatography–mass spectrometry (GC–MS). By integrating these analytical techniques with multivariate statistical analysis, we seek to provide a deeper understanding of the biochemical basis for the unique flavors of these two traditional hams. The findings of this study will not only contribute to the scientific understanding of flavor formation in dry-cured hams but also provide valuable insights for optimizing processing techniques to enhance product quality.

## 2 Materials and methods

### 2.1 Materials

Six 10-month-old hybrid pigs with Wujin pig bloodline were provided by Xuanwei Shunda Ham Co., Ltd., while six Jinhua pigs of 10-month-old were provided by Jinzi Ham Co., Ltd. The 2,2-dimethyl-2-silapentane-5-sulfonate (DSS) standard solution was purchased from Anachro Technologies Inc. (Calgary, AB, Canada). 2,6-Di-tert-butyl-4-methylphenol (BHT) standard solution was obtained from Sinopharm Chemical Reagent Co., Ltd. Methanol and hexane (HPLC-grade) were purchased from Tedia Co. (Fairfield, OH, USA). All aqueous solutions were prepared using ultrapure water produced using a Milli-Q system (18.2 Ωcm; Mil-lipore, Bedford, MA, USA). All other chemicals used in NMR experiments were HPLC-grade.

### 2.2 Processing of ham and sample preparation

Twelve hind legs from these two breeds of pigs were provided by Xuanwei Shunda Ham Co. Ltd. and Jinzi Ham Co. Ltd. respectively, and both companies ensured that the hind legs were sourced from pig breeds of the same age, batch, and similar weight. In addition, the companies also ensured that the breeding and slaughtering methods of the pig breeds were in accordance with the ethical guidelines for animal research. Six of the hind legs were processed into hams according to the company's traditional techniques. At the same time, samples were collected from the biceps femoris of the other 6 hind legs, vacuum packed and stored at −80 °C for analysis. The processing of ham involved various steps such as trimming the raw material legs, salting, surface cleaning, hanging and air drying, fermentation and maturation. The entire processing cycle was two years.

Xuanwei ham uses a dry curing method, with the amount of sea salt used being 6–7 % (*w*/w) of the leg weight, and it is layered cured for 15–20 days at an environmental temperature of 4–10 °C. Jinhua ham also uses a dry curing method, but with a higher salt content (8–10 % w/w) and a longer curing period (25–35 days), with temperatures ranging from 5 to 10 °C. After curing, both hams are stacked at 2–5 °C for 15–20 days to ensure even distribution of salt within the muscle. The surface salt crust is thoroughly brushed off and the hams are washed with warm water, then shaped into their characteristic forms. Xuanwei ham is then air-dried and fermented in a well-ventilated curing room at 10–18 °C and 60–75 % relative humidity (RH) for 6–8 months. Jinhua ham undergoes a unique high-temperature fermentation stage: after initial drying, it is fermented for 2–3 months during late spring to early summer at 25–30 °C and 75–85 % RH, followed by a period of aging. After processing was completed, the biceps femoris part of the finished ham was taken as a sample, and then the small molecular metabolites and FFAs of the intramuscular fat and subcutaneous fat were determined, respectively.

### 2.3 Monitoring changes in processing temperature and relative humidity

The Temperature & Humidity Meter (TP500V2, Toprie, China) were installed separately in the processing workshops of Xuanwei Shunda Ham Co., Ltd. and Jinzi Ham Co., Ltd. Starting from the salting process of ham, the temperature and relative humidity of the workshop were recorded in real-time every 2 h, and the average value of the month was taken as the temperature and relative humidity value for that month. The trend chart of temperature and relative humidity changes during ham processing and fermentation was plotted.

### 2.4 GC–MS analysis of FFA composition

Determination of FFA composition was done according to the method of our previous study (Shi et al., 2018) with modifications. Briefly, the FFAs were derivatized into their corresponding fatty acid methyl esters (FAMEs) via acid-catalyzed esterification. For the analysis of FFAs in the subcutaneous fat of ham, a 5 mg freeze-dried sample was accurately weighed and added with 2 mL of a solution of 5 % (*v*/v) sulfuric acid in anhydrous methanol. 100 μL of 1 mg/mL undecanoic acid triglyceride (C11:0) in methanol was added as an internal standard. Then, an aliquot of 25 μL butylated hydroxyltoluene methanol solution (0.2 %, m/v) was added to inhibit oxidation. The mixture was vortexed for 1 min and then heated for 1.5 h at 90 °C to complete the methylation reaction. After cooling to room temperature, 1 mL of n-hexane and 2 mL of a saturated sodium chloride solution were added for liquid-liquid extraction of the FAMEs. The vial was vortexed for 60 s and centrifuged at 3500 rpm for 5 min at 4 °C. Subsequently, 100 μL of the upper organic phase (n-hexane layer) containing the FAMEs was taken for GC–MS analysis. The parameter conditions for GC–MS analysis are shown in Table 1. For the determination of FFAs in the intramuscular fat, 10 mg of freeze-dried lean ham sample was accurately weighed to account for its lower fat content and to ensure the absolute amount of analytes was within the optimal range for robust quantification. Other conditions were the same as above.

**Table 1:** GC–MS measurement parameters.

Project	Parametric
Sample Injection Volume	1 μL
Split Injection Mode	Split Mode (60:1)
Septum Purge Flow Rate	0.2 mL/min
Carrier Gas	Helium
Chromatographic Column	Agilent DB-2259 (10 m × 0.1 μm × 0.1 μm)
Column Pressure	46 psi
Column Oven Temperature Ramp Program	Hold at 55 °Cfor 1 min, increase to 205 °C at 30 °C/min for 1 min, increase to 210 °C at 5 °C/min for 1 min.
Front Injector Port Temperature	250 °C
Transfer Line Temperature	230 °C
Ion Source Temperature	230 °C
Quadrupole Temperature	150 °C
Ionization Voltage	−70 eV
Mass Range	30–400 *m*/*z*
Scanning Mode	Scan/SIM
Solvent Delay Time	1 min

### 2.5 NMR analysis of small molecular metabolites

The analysis of small molecular metabolites was based on previous research methods with slight modifications (Xiao, Luo, et al., 2019). An amount of 50 mg of freeze-dried fat and lean meat powder was accurately weighed, added with 1000 μL of pure water, vortexed for 1 min, then sonicated in an ice water bath for 32 s, followed by centrifugation at 13000 rpm for 10 min at 4 °C. 800 μL of supernatant was taken out and filtered by centrifugation with ultrafiltration membrane for 40 min. 450 μL of filtrate was taken out and placed in a newly numbered centrifuge tube. 50 μL of DSS reagent was added and vortexed for 10 s, followed by centrifugation at 13000 rpm for 2 min at 4 °C. 480 μL of the sample was loaded into an NMR tube for NMR spectrum analysis.

The concentration of the internal standard, 2,2-dimethyl-2-silapentane-5-sulfonate (DSS), in the final NMR sample was 500 μM. This was achieved by adding 50 μL of a 10 mM DSS stock solution (prepared in D₂O) to 450 μL of the filtered sample supernatant, resulting in a total volume of 500 μL.

Metabolite concentrations were quantified relative to the DSS internal standard. The methyl proton signal of DSS (δ 0.00 ppm) was used as the chemical shift reference and, crucially, for quantification due to its known concentration and sharp singlet peak. The concentration of each metabolite was calculated automatically during spectral profiling using the Chenomx NMR Suite software (version 8.6, Chenomx Inc., Canada) and cross-verified manually using the following fundamental equation:

$$C_{\mathrm{met}=\frac{A_{\mathrm{met}}N_{\mathrm{DSS}}}{A_{\mathrm{DSS}}N_{\mathrm{met}}}C_{\mathrm{DSS}}\times \frac{{\mathrm{MW}}_{\mathrm{met}}}{{\mathrm{MW}}_{\mathrm{DSS}}}}$$

C_met_: concentration of the metabolite (in mM or μmol/g).

A_met_: integrated area of the selected proton signal of the metabolite.

A_DSS_: ntegrated area of the DSS methyl proton signal (at δ 0.00 ppm).

N_DSS_: number of protons contributing to the DSS methyl signal (9 protons).

N_met_: number of protons contributing to the selected signal of the metabolite.

C_DSS_: known concentration of DSS in the NMR tube (500 μM).

MW_met_: molecular weight of the metabolite.

MW_DSS_: molecular weight of DSS (218.33 g/mol).

The final concentrations are reported in millimolar (mM) units within the NMR sample and were subsequently normalized to the dry weight of the original tissue sample (μmol/g dry weight) for all statistical analyses and comparisons. This methodology provides absolute quantification of metabolite concentrations.

The specific parameters for NMR data acquisition were as follows: temperature of 298.15 K, nuclear magnetic resonance frequency of 600.20 MHz, transient/scan of 128, frequency domain size of 131,072, spectral width of 8403.36, time domain size of 65,536, pulse sequence: noesygppr1d.

### 2.6 Statistical analysis

Excel 2019 was used to compile the preliminary data on FFAs detected by GC–MS, and SPSS 26.0 was used to analyze the data. Comparisons with means were performed with *t*-test analysis and differences were regarded as significant at *P* < 0. 05. After being normalized in Excel 2019, extracted small molecular metabolites were arranged into a two-dimensional data matrix. Partial least squares discriminant analysis (PLS-DA) were two multivariate statistical analyses performed using Metabo Analyst 5.0 software (Yang et al., 2022). The significant variations in small molecular metabolites between Xuanwei ham and Jinhua ham were ascertained by using statistical display criteria and analysis of variance (student's *t*-test) based on the significance of the projected variables (VIP) (*P*-value). There were six replicates for all indicators in each group.

## 3 Results and discussion

### 3.1 Analysis of temperature and relative humidity in the processing workshops of Xuanwei and Jinhua

**Fig. 1:**
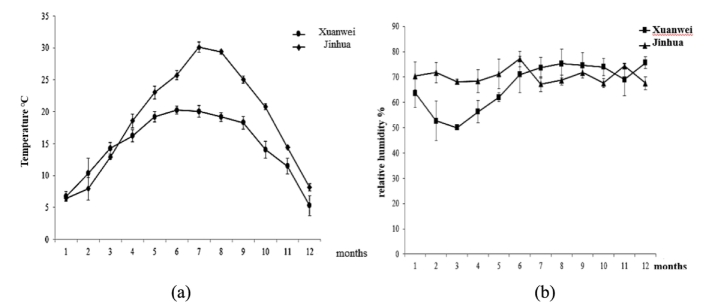
Monthly average temperature (a) and relative humidity (b) in Xuanwei and Jinhua regions.

The processing of Chinese hams is fundamentally analogous to that of other dry-cured ham products, comprising a series of interconnected stages, including curing, washing, drying, fermentation, maturation and post-maturation. The principal factors influencing the production of diverse flavor profiles in dry-cured hams are variations in temperature, humidity and fermentation maturation time (Hu et al., 2023). As illustrated in Fig. 1, the variation in temperature and humidity during the curing and maturation of Xuanwei and Jinhua hams throughout the year is characterized, with the values for each month representing the average of temperature and humidity over the two-year period. As demonstrated in Fig. 1a, the variation of temperature and humidity before March, the temperatures in Xuanwei and Jinhua were low, which was conducive to the curing and dehydration of the ham and belonged to the “low-temperature dehydration stage”; after March, the temperature rise rate in Jinhua increased faster, with temperatures between 15 and 30 °C, which belonged to the “medium-temperature fermentation stage”. The temperature reached its peak in July and August, at approximately 30 °C, which is conducive to the fermentation of Jinhua ham, improves the enzyme vitality of the ham, promotes the degradation of ham proteins and fats, and generates volatile flavor substances, so that the ham produces a strong aroma (Xiong et al., 2022). High-temperature curing represents a distinctive process in the production of Jinhua ham, with this method being instrumental in generating its distinctive aromatic flavor (Deng et al., 2022). This characteristic, coupled with the relatively modest increase in temperature during the curing process in Xuanwei, underscores the pivotal role of high-temperature curing in shaping the unique sensory profile of Jinhua ham. The period from June to July sees the temperature range reach its zenith, with a range of 16 to 18 °C. This is primarily attributable to Xuanwei's high-altitude location, which results in a distinct reduction in temperature. The lower temperatures in this region lead to a decline in microbial vitality and enzyme activity, contributing to the unique characteristics of Xuanwei ham. The temperature rise and duration exert a significant influence on the degree of hydrolysis of fats and proteins, thereby affecting the formation of the ham's flavor. Correlation exists between temperature and humidity, with the former being influenced by the prevailing local climate. Humidity, being equally crucial, impacts the development of flavor and the quality of the ham. The influence of humidity is twofold: firstly, it affects the rate of water loss from the ham, and secondly, it impacts the change in its own water activity. Additionally, it has a role to play in the growth of micro-organisms. As demonstrated in Fig. 1b, relative humidity in Xuanwei is lower than that in Jinhua before June. This is due to the higher humidity in Jinhua being a consequence of the higher relative humidity during the long cloudy and rainy weather in spring. This prolonged period of low to medium temperature dehydration and fermentation results in the surface of the ham being susceptible to the growth of various molds, which are then gradually covered by the dominant bacteria, thereby forming a microbial barrier for the fermentation process. During this period, the microbial barrier of internal fermentation is formed, with muscle proteins and fats being degraded under the action of endogenous enzymes, producing degradation products such as peptides, free amino acids and FFAs (Huan et al., 2005). These, in turn, promote further oxidation, Strecker degradation and Maillard reactions of the ham, leading to the formation of bioreactors with characteristic flavor compounds (López-Pedrouso et al., 2019). The significant decrease in humidity in Xuanwei during the months of March and April is due to the low rainfall and strong winds in spring, resulting in low humidity (Yan et al., 2005). From July to October, Xuanwei had higher relative humidity than Jinhua, mainly due to the higher summer and autumn temperatures in Jinhua, which resulted in lower humidity (López-Pedrouso et al., 2019). The elevated relative humidity in Xuanwei fosters the growth of mold on the surface of the ham, leading to the formation of “green hairs”. According to Li (Li et al., 2022), these green molds are mainly composed of specific beneficial strains from the Penicillium and Aspergillus genera. The study also found that Penicillium is the core of the surface fungal community and is significantly positively correlated with the free amino acid and volatile flavor compound content of ham. These hairs further stimulate the proliferation of micro-organisms, resulting in the production of enzymes that contribute to the ham's distinctive qualities, namely tenderness, saltiness, and lightness, while maintaining a low greasy texture. In contrast, Jinhua experiences consistently higher levels of relative humidity throughout the year, factor that significantly contributes to the strong flavor profile of Jinhua ham.

### 3.2 Analysis of free FFA acids

The FFA composition is a critical determinant of the final quality and flavor potential of dry-cured hams (Liu et al., 2023). Our analysis of subcutaneous (SF, Table 2) and intramuscular fat (IMF, Table 3) reveals fundamental and tissue-specific lipid compositional differences between the raw legs destined for Xuanwei (XW) and Jinhua (JH) ham production. Analysis of subcutaneous (SF, Table 2) and intramuscular fat (IMF, Table 3) reveals fundamental differences in the raw materials.JH raw legs exhibit significantly higher total FFA content (414.02 vs. 314.25 μg/mg) and elevated PUFA levels (∑PUFA: 69.77 vs. 38.62 μg/mg) in subcutaneous fat compared to XW, resulting in a higher PUFA/SFA ratio (0.51 vs. 0.39). Key flavor precursors like linoleic acid (C18:2n6c) and arachidonic acid (C20:4n6) are 1.7–1.9 times more abundant in JH SF·In contrast, IMF composition is highly similar between types, with no significant differences in total FFA, ∑SFA, ∑MUFA, ∑PUFA, or PUFA/SFA ratio.This indicates JH's raw material possesses a richer subcutaneous PUFA reservoir, potentially contributing to more intense flavor development during processing, while IMF provides a comparable base for taste formation.

**Table 2:** The results of free fatty acid content in subcutaneous fat of Xuanwei ham raw meat and Jinhua ham (μg/mg).

Free fatty acid	Xuanwei ham	Jinhua ham
(C10:0)	0.73 ± 0.06	0.73 ± 0.07
(C12:0)	0.60 ± 0.06^a^	0.82 ± 0.07^b^
(C14:0)	6.46 ± 1.04	8.04 ± 1.08
(C15:0)	0.29 ± 0.03^a^	0.49 ± 0.05^b^
(C16:0)	68.93 ± 1.69^a^	92.94 ± 1.38^b^
(C16:1n7)	9.15 ± 1.85	12.06 ± 2.12
(C17:0)	1.38 ± 0.05^a^	2.35 ± 0.26^b^
(C17:1n7)	1.14 ± 0.15^a^	1.78 ± 0.23^b^
(C18:0)	21.66 ± 1.66^a^	30.21 ± 4.81^b^
(C18:1n9c)	158.60 ± 30.88	186.88 ± 30.35
(C18:2n6c)	28.53 ± 1.83^a^	49.81 ± 8.33^b^
(C18:3n3)	3.00 ± 0.32^a^	5.84 ± 0.67^b^
(C20:0)	0.65 ± 0.09	0.74 ± 0.09
(C20:1)	5.24 ± 0.97	6.41 ± 0.88
(C20:2)	2.95 ± 0.43^a^	6.12 ± 0.79^b^
(C20:3n6)	0.66 ± 0.06^a^	1.43 ± 0.17^b^
(C20:4n6)	2.77 ± 0.45^a^	5.13 ± 0.68^b^
(C20:3n3)	0.70 ± 0.12^a^	1.44 ± 0.18^b^
(C22:0)	0.26 ± 0.03	0.26 ± 0.01
(C22:1n9)	0.54 ± 0.05	0.52 ± 0.01
(∑SFA)	100.97 ± 12.53^a^	136.59 ± 21.79^b^
(∑MUFA)	174.67 ± 33.60	207.66 ± 33.49
(∑PUFA)	38.62 ± 2.11^a^	69.77 ± 10.66^b^
(FFA)	314.25 ± 47.81^a^	414.02 ± 65.92^b^
PUFA/SFA	0.39 ± 0.03^a^	0.51 ± 0.01^b^

Note: Values with the different lowercase letters within the same row mean significant differences (*P* < 0.05).

**Table 3:** The results of free fatty acid content in intramuscular fat of Xuanwei ham raw meat and Jinhua ham (μg/mg).

Free fatty acid	Xuanwei ham	Jinhua ham
(C10:0)	0.49 ± 0.10^b^	0.37 ± 0.02^a^
(C12:0)	0.37 ± 0.06	0.34 ± 0.03
(C14:0)	3.26 ± 0.41	3.06 ± 0.30
(C15:0)	0.19 ± 0.02	0.20 ± 0.02
(C16:0)	41.79 ± 4.82	41.04 ± 7.29
(C16:1n7)	6.44 ± 1.00	5.81 ± 0.84
(C17:0)	0.97 ± 0.09	1.02 ± 0.12
(C17:1n7)	0.68 ± 0.14	0.59 ± 0.06
(C18:0)	14.22 ± 0.73	15.09 ± 2.81
(C18:1n9c)	90.38 ± 13.93	75.38 ± 15.13
(C18:2n6c)	14.42 ± 0.51	15.10 ± 2.99
(C18:3n3)	1.31 ± 0.32	1.33 ± 0.21
(C20:0)	0.49 ± 0.08	0.44 ± 0.04
(C20:1)	3.49 ± 0.57^b^	2.27 ± 0.29^a^
(C20:2)	1.59 ± 0.39	1.55 ± 0.27
(C20:3n6)	0.83 ± 0.06	0.80 ± 0.05
(C20:4n6)	14.64 ± 0.82	14.28 ± 0.77
(C20:3n3)	0.54 ± 0.05	0.55 ± 0.03
(C22:0)	0.22 ± 0.02	0.23 ± 0.02
(C22:1n9)	0.31 ± 0.00^b^	0.28 ± 0.01^a^
(∑SFA)	61.99 ± 6.09	61.79 ± 10.57
(∑MUFA)	101.30 ± 15.55	84.32 ± 16.29
(∑PUFA)	33.34 ± 1.31	33.60 ± 3.00
(FFA)	196.64 ± 21.90	179.71 ± 29.82
PUFA/SFA	0.54 ± 0.06	0.55 ± 0.04

Note: Values with the different lowercase letters within the same row mean significant differences (*P* < 0.05).

As shown in Table 4, the FFA content and PUFAlevel in the SF of Xuanwei ham were significantly higher than those in Jinhua ham (601.71 vs. 528.37 μg/mg and 102.69vs. 76.09 μg/mg, respectively; *P* < 0.05). Key flavor precursors such as linoleic acid (C18:2n6c) and arachidonic acid (C20:4n6) were particularly elevated. Consequently, the PUFA/SFA ratio of Xuanwei ham (0.51 μg/mg) was significantly higher than that of Jinhua ham (0.38 μg/mg), indicating a nutritionally superior lipid profile. Additionally, Xuanwei ham contained higher levels of medium- and short-chain saturated fatty acids (e.g., C10:0–C15:0), which are more susceptible to oxidative degradation and may contribute more directly to the distinct flavor formation (Wu et al., 2020).

**Table 4:** The results of free fatty acid content in subcutaneous fat of Xuanwei Ham and Jinhua Ham (μg/mg).

Free fatty acid	Xuanwei ham	Jinhua ham
C10:0	1.00 ± 0.14^b^	0.56 ± 0.11^a^
C12:0	0.79 ± 0.11^b^	0.53 ± 0.09^a^
C14:0	9.66 ± 1.24^b^	6.92 ± 0.78^a^
C15:0	0.49 ± 0.08^b^	0.37 ± 0.02^a^
C16:0	139.90 ± 18.38	132.97 ± 9.30
C16:1n7	22.25 ± 6.29	15.61 ± 2.32
C17:0	1.99 ± 0.41	1.53 ± 0.22
(C17:1n7)	1.59 ± 0.38	1.19 ± 0.36
C18:0	49.03 ± 5.98	54.11 ± 10.77
C18:1n9c	266.78 ± 37.42	232.82 ± 27.04
C18:2n6c	88.45 ± 7.59^b^	63.50 ± 15.54^a^
C18:3n3	4.71 ± 1.38	5.81 ± 4.21
C20:0	0.49 ± 0.01	0.59 ± 0.14
C20:1	4.74 ± 0.32	4.78 ± 0.67
C20:2	4.84 ± 1.07	3.88 ± 0.86
C20:3n6	0.94 ± 0.16	0.74 ± 0.17
C20:4n6	3.64 ± 0.30^b^	2.01 ± 0.44^a^
C20:3n3	0.12 ± 0.03	0.15 ± 0.07
C22:0	0.06 ± 0.01	0.06 ± 0.01
C22:1n9	0.25 ± 0.02	0.25 ± 0.01
∑SFA	203.40 ± 21.82	197.64 ± 21.22
∑MUFA	295.61 ± 43.93	254.64 ± 29.91
∑PUFA	102.69 ± 10.00^b^	76.09 ± 13.45^a^
FFA	601.71 ± 60.10	528.37 ± 29.00
PUFA/SFA	0.51 ± 0.10^b^	0.38 ± 0.04^a^

Note: Values with the different lowercase letters within the same row mean significant differences (*P* < 0.05).

In Table 5, although the total FFA, SFA, and MUFA contents did not differ significantly between the two hams, the PUFA/SFA ratio of Xuanwei ham remained significantly higher (0.50 vs. 0.29 μg/mg). It is noteworthy that the content of arachidonic acid (C20:4n6) was also significantly greater in the intramuscular fat of Xuanwei ham, further supporting its nutritional advantage. The drastic decline in the PUFA/SFA ratio in Jinhua ham, in stark contrast to its stability in Xuanwei ham, highlights a fundamental difference in the oxidative stress environment and antioxidant-prooxidant balance during processing. This disparity is likely driven by two key factors:

**Table 5:** The results of free fatty acid content in intramuscular fat of Xuanwei ham and Jinhua ham (μg/mg).

Free fatty acid	Xuanwei ham	Jinhua ham
C10:0	0.17 ± 0.06	0.17 ± 0.04
C12:0	0.15 ± 0.04	0.14 ± 0.03
C14:0	1.50 ± 0.56	1.52 ± 0.47
C15:0	0.16 ± 0.01	0.16 ± 0.02
C16:0	29.83 ± 11.11	37.21 ± 14.32
C16:1n7	4.38 ± 2.13	4.47 ± 2.07
C17:0	0.44 ± 0.08	0.42 ± 0.11
C17:1n7	0.26 ± 0.06	0.25 ± 0.07
C18:0	12.76 ± 3.21	16.71 ± 4.91
C18:1n9c	50.39 ± 18.36	56.37 ± 26.29
C18:2n6c	15.06 ± 2.14	12.34 ± 4.80
C18:3n3	0.97 ± 0.08	0.77 ± 0.18
C20:0	0.12 ± 0.03	0.17 ± 0.05
C20:1	0.87 ± 0.13	0.97 ± 0.30
C20:2	0.59 ± 0.07	0.54 ± 0.16
C20:3n6	0.27 ± 0.01^b^	0.18 ± 0.07^a^
C20:4n6	4.11 ± 0.75^b^	2.26 ± 0.81^a^
C20:3n3	0.04 ± 0.00	0.03 ± 0.00
C22:0	0.05 ± 0.01	0.05 ± 0.01
C22:1n9	0.13 ± 0.01	0.14 ± 0.01
∑SFA	45.19 ± 15.00	56.56 ± 19.80
∑MUFA	56.03 ± 20.63	62.20 ± 28.70
∑PUFA	21.03 ± 1.51	16.13 ± 5.70
FFA	122.25 ± 37.07	134.88 ± 52.97
PUFA/SFA	0.50 ± 0.15^b^	0.29 ± 0.05^a^

Note: Values with the different lowercase letters within the same row mean significant differences (*P* < 0.05).

The pig breed is a primary determinant. Jinhua pigs are renowned for their high intramuscular fat (IMF) content, a trait often inversely correlated with the muscle's antioxidant enzyme activity. Conversely, the hybrid pigs with Wujin bloodline used for Xuanwei ham may possess a different antioxidant profile. More crucially, the significantly higher initial content of polyunsaturated fatty acids (PUFAs) in the subcutaneous fat of Xuanwei raw legs (as reported in Table 2) suggests that the pigs' diet or genetics might also provide a richer pool of lipid-soluble antioxidants (e.g., Vitamin E) to protect these vulnerable fats from oxidation. The higher PUFA content itself could be an indicator of a better antioxidant system in the raw material. (Song et al., 2023) The high-temperature fermentation (∼30 °C) undergone by Jinhua ham acts as a powerful external pro-oxidant. Temperature exponentially accelerates the kinetics of lipid oxidation. This intense thermal stress likely overwhelms the intrinsic antioxidant defenses, leading to the massive depletion of PUFAs observed. In contrast, the cooler and more stable processing conditions of Xuanwei ham minimize thermal-driven oxidation, allowing the natural antioxidant systems (both endogenous and any from the diet) to better preserve the PUFAs. (Hernández-Correas et al., 2024).

While this study did not quantify specific antioxidant compounds (e.g., Vitamin E, glutathione), the PUFA/SFA ratio is a robust integrated measure of the net oxidative outcome. The dramatic difference in this ratio between the two hams leads us to conclude that the pro-oxidant forces (driven by high temperature) in Jinhua ham far exceeded its antioxidant capacity (Zhu et al., 2014), whereas in Xuanwei ham, the antioxidant-prooxidant balance was maintained, resulting in superior oxidative stability and PUFA retention.

Table 4, Table 5 present the FFA composition, as well as the intramuscular and subcutaneous FFA content, of the two hams. A total of 20 FFA were identified in both hams, with oleic acid, palmitic acid, linoleic acid, and stearic acid exhibiting the highest concentrations. Between the two hams, there were notable variations (*P* < 0.05) in the levels of several intramuscular and subcutaneous FFA. The intramuscular unsaturated fatty acid content of Xuanwei ham accounted for 63.03 % of the FFAs, and the subcutaneous unsaturated fatty acid content accounted for 66.19 % of the FFAs. content of Jinhua ham accounted for 58.07 % and 62.59 % of the FFAs, respectively, which indicated that the FFAs of Xuanwei ham had a higher proportion of unsaturated fatty acids than that of Jinhua ham, which had higher antioxidant activity. Our results showed that the total content of subcutaneous polyunsaturated fatty acids (PUFA), as well as linoleic acid (C18:2n6c), was significantly higher in Xuanwei ham (XW) than in Jinhua ham (JH) (*P* < 0.05). However, an intriguing finding was that the higher PUFA retention in XW likely does not solely originate from the raw material but, more importantly, indicates a less pronounced extent of lipid oxidation during its processing (Zhang et al., 2019). It is noteworthy that while the FFA profile and ratio changes indirectly suggest differential oxidation, this study did not directly quantify volatile oxidation products, such as aldehydes and ketones. Quantitative analysis of these key flavor compounds and oxidative markers will be a crucial objective for future research to provide a more comprehensive elucidation of the divergent flavor formation mechanisms between these two traditional hams.

Beyond the significant differences in specific FFAs, an analysis of the distribution patterns of the four dominant FFAs (palmitic, stearic, oleic, and linoleic acids) reveals a consistent hierarchy in their relative abundance across both tissue types and ham varieties: Oleic acid (C18:1) > Palmitic acid (C16:0) > Stearic acid (C18:0) > Linoleic acid (C18:2). This pattern aligns with the fundamental biochemistry of lipid deposition in pigs. However, the divergent roles of SF and IMF in flavor formation are underscored by the differences in their absolute composition and stability. The subcutaneous fat, with its higher total lipid content and greater exposure to oxygen and microbial activity, serves as the primary reservoir for volatile flavor precursors (Zhang et al., 2023). The significantly higher PUFA content and PUFA/SFA ratio we observed in Xuanwei SF imply a greater potential for oxidative generation of aldehydes, alcohols, and ketones responsible for aroma, albeit at a different rate and profile due to Xuanwei's milder processing conditions. Conversely, the intramuscular fat is intricately woven within the muscle matrix. Its composition, particularly the higher IMF content inherent to Jinhua pigs, is critically linked to textural properties (e.g., tenderness, juiciness) and the sustained release of non-volatile taste compounds during mastication. The lipids in IMF also undergo lipolysis, releasing FFAs that contribute directly to taste (e.g., soapy, pungent) or serve as substrates for further flavor development within the muscle (Tyra et al., 2013). Therefore, the consistent relative pattern confirms biological predictability, while the absolute quantitative differences between tissues and breeds underpin the mechanistic divergence in how subcutaneous and intramuscular fats contribute to the overall flavor profile: SF primarily dictating the aroma volatile spectrum and IMF profoundly influencing texture and taste release.

While our study did not establish a quantitative kinetic model, the stark contrast in PUFA retention provides strong indirect evidence that temperature is a primary driver of FFA degradation rates. This correlation is strongly supported by existing literature. For instance, Zhang et al. (2005) directly reported that diene value and carbonyl value—key indicators of lipid oxidation—exhibited a highly significant positive correlation with temperature during Jinhua ham processing, with determination coefficients (R^2^) as high as 0.9388 and 0.8416, respectively. Therefore, the high-temperature stage is not merely a cultural characteristic but a critical biochemical accelerator that shapes the distinct volatile flavor profile of Jinhua ham through rapid lipid oxidation.

### 3.3 ^1^H NMR spectra of small molecular metabolites in ham

The utilization of nuclear magnetic resonance (NMR) spectroscopy for the expeditious identification and measurement of metabolites confers a number of distinct advantages. On-volatile constituents such as amino acids and nucleotides present in raw meat are of paramount importance in the development of meat flavor, despite the significance of lipid oxidation and the Maillard reaction (Mena et al., 2012). The taste, flavor, softness, and juiciness of meat and meat products can all be influenced by these nonvolatile flavor precursors, which include sugars, amino acids, creatine, carnosine, and nucleotides (Tian et al., 2022). As demonstrated in Tables S1 and S2, a total of 60 metabolites were identified in raw legs and 55 metabolites were identified in ham, including 18 amino acids, 4 derivatives, 13 organic acids, 4 nucleosides, and 1 sugar. ic acids, 4 alcohols, 4 vitamins, 2 peptides, 1 sugar, and 10 other metabolites. The results obtained in this study were similar to those reported in previous study (Ding et al., 2021).

The reduction in the number of detectable metabolites from 60 in raw legs to 55 in cured hams reflects the dynamic biochemical transformations occurring during processing. Specific metabolites that decreased below the detection limit include glutamine, asparagine, glutathione, inosine, and 2-hydroxybutyrate. This reduction is not merely a loss but primarily a result of their active participation in flavor-forming pathways: The most notable example is inosine, which is degraded to hypoxanthine (whose content increases from raw to ham, as shown in Table 6、7) by endogenous enzymes. Similarly, creatine (not among the 5 lost but significantly reduced) decomposes into creatinine and further to sarcosine and urea (Zhang, Wang, et al., 2025). Glutamine and asparagine are crucial amino acids that can undergo deamidation to form their corresponding acids (glutamate and aspartate) or serve as key participants in the Maillard reaction and Strecker degradation, generating a plethora of volatile aroma compounds and taste substances (Rueda et al., 2020). Glutathione, a tripeptide, is likely hydrolyzed into its constituent amino acids (glutamate, cysteine, glycine), which then enter other reaction pathways. (Kulczyński et al., 2019).

**Table 6:** Contents of six small molecular metabolites of Xuanwei ham raw meat and Jinhua ham raw meat (mg/g).

Small molecular metabolites	Raw Meat	
	Xuanwei	Jinhua
Lactate	32.95 ± 0.92^b^	21.51 ± 0.57^a^
Creatine	20.09 ± 1.23^b^	16.73 ± 0.10^a^
Taurine	2.20 ± 0.65^b^	1.02 ± 0.01^a^
Anserine	18.54 ± 0.41	17.55 ± 0.91
Hypoxanthine	0.35 ± 0.05^a^	1.51 ± 0.14^b^
Inosine	1.86 ± 0.93	2.05 ± 0.09

Note: Values with the different lowercase letters within the same row mean significant differences (*P* < 0.05).

The extent of these transformations is profoundly influenced by processing conditions. The higher temperature and humidity experienced by Jinhua ham, particularly during its distinctive fermentation stage, significantly accelerate the reaction rates of these degradation and conversion processes compared to the cooler and drier conditions of Xuanwei ham. This results in a more pronounced decrease in the levels of these precursor metabolites in Jinhua ham, ultimately contributing to its stronger aroma profile.

Utilizing ^1^H NMR mapping and metabolite attribution detection, a total of six water-soluble compounds were identified as having a significant impact on the flavor quality of ham. As illustrated in Table 6, the levels of lactic acid and creatine were found to be higher in the two raw legs, exhibiting a notable difference between them (*P* < 0.05). This discrepancy can be attributed to the various stages of the slaughtering process for the raw pigs. The deacidification process of the meat at the later stage, and at the same time, the lactic acid and creatine would also be further degraded and transformed in the later stage of processing and storage, which would have a certain effect on the formation of the flavor. The dramatic shift in the metabolite profile from raw legs (dominated by lactic acid, creatine, anserine) to cured hams (dominated by free amino acids like glutamate and lysine) unequivocally points to intense microbial and enzymatic activity during processing (Zhu et al., 2021). While the current study focused on the metabolomic endpoint and did not quantify microbial communities, the observed changes are entirely consistent with well-established roles of microorganisms in cured meat fermentation (Li et al., 2022). The decline in lactic acid, initially abundant from post-mortem glycolysis, is indicative of its utilization as a carbon source by certain microbial populations. The significant degradation of creatine and anserine is primarily driven by endogenous muscle enzymes (e.g., creatinase, carnosinase), whose activity can be modulated by the changing microbial environment and salt content (Rutigliano et al., 2023). Conversely, the massive accumulation of free amino acids, particularly glutamic acid and lysine, is a hallmark of proteolysis. This process is catalyzed by both endogenous meat proteases (e.g., cathepsins, calpains) and exogenous proteases secreted by the complex microbial community that develops on the ham surface (Li, Zhou, et al., 2024). Lactic acid bacteria (LAB) and coagulase-negative staphylococci (CNS), which are dominant in these ecosystems, are renowned for their proteolytic and peptidolytic activities (Tian et al., 2023; Zhou et al., 2023). They break down muscle proteins into peptides and ultimately into free amino acids, which are the direct precursors for the Maillard reaction and Strecker degradation, forming the critical volatile and non-volatile flavor compounds (Castellano et al., 2016). The specific microbial consortia on Xuanwei and Jinhua hams, shaped by their distinct temperature and humidity profiles, likely contribute to the differential quantitative patterns of amino acid release observed between the two products (Gong et al., 2023). Therefore, the microbial community acts not merely as a passive spectator but as a crucial biochemical engine that drives the transformative reactions responsible for the development of the characteristic ham flavor(Zhu et al., 2021). Taurine has a variety of physiological functions and is an essential nutrient for human health (Roşca et al., 2022). The taurine content in Xuanwei raw leg was found to be approximately 2.24 times higher than that observed in Jinhua raw leg (*P* < 0.05). As demonstrated in Table 6、7, both types of hams exhibited higher levels of lactic acid and anserine, thereby exerting a more substantial influence on the ham's flavor profile. In comparison with Jinhua ham, the content of taurine in Xuanwei ham was found to be 76.7 % higher (*P* < 0.05). Taurine, an essential nutritional amino acid, possesses specific physiological functions. Consequently, the nutritional quality of the two hams differed, primarily due to variations in the raw material leg and the processing technology (Pérez-Santaescolástica et al., 2019).

Firstly, the genetic background of the pig breeds, as the markedly higher initial content in XW raw legs suggests a possible breed-specific characteristic in taurine metabolism or accumulation in hybrid Wujin pigs; secondly, differential stability during processing, where the cooler and drier processing conditions of XW ham may more effectively inhibit microbial-mediated degradation of taurine, leading to its better preservation. Regarding flavor implications, the significant difference in taurine content may contribute to the overall flavor profiles of the two hams. Although taurine has a relatively high taste threshold, it is recognized as an effective umami enhancer and taste modulator. It can synergize with umami amino acids like glutamate to enhance the savory perception and mitigate salty and bitter tastes, thereby promoting a mellow and continuous flavor profile (Ando, 2020). In our study, the concomitantly higher levels of both taurine and glutamate in XW ham (Table 7) may well be intrinsically linked to its traditionally described ‘light and delicate’ flavor with a lingering aftertaste. In contrast, JH ham develops its ‘strong and robust’ aroma dominated by volatile compounds (e.g., ketones, aldehydes) derived from more intense lipid oxidation and Maillard reactions. Thus, taurine, as a key component of non-volatile taste substances, could be an important factor differentiating the flavor contours of these two traditional dry-cured hams (Zheng et al., 2025).

**Table 7:** Contents of six small molecular metabolites of Xuanwei Ham and Jinhua Ham (mg/g).

small molecular metabolites	Ham	
	Xuanwei	Jinhua
Lactate	17.03 ± 5.63	14.53 ± 5.66
Creatine	5.47 ± 1.07	4.06 ± 1.12
Taurine	1.66 ± 0.19^b^	0.94 ± 0.35^a^
Anserine	13.00 ± 3.22	10.93 ± 1.36
Hypoxanthine	1.91 ± 0.36	2.02 ± 0.14
Inosine	1.09 ± 0.33	0.74 ± 0.03

Note: Values with the different lowercase letters within the same row mean significant differences (*P* < 0.05).

As demonstrated in Table 6, Table 7, the processing of Xuanwei ham resulted in a significant decrease in the contents of lactic acid, creatine, and anserine, with reductions of 43.82 % (*P* < 0.05), 72.77 % (*P* < 0.05), and 29.88 % (*P* < 0.05), respectively. In addition, the levels of la In contrast, the content of lactic acid, creatine, and anserine in Jinhua ham decreased by 53.89 % (*P* < 0.05), 15.96 % (*P* < 0.05), and 15.96 % (*P* < 0.05), respectively (*P* < 0.05) and 37.72 % (*P* < 0.05), respectively. The decrease in creatine content may be decomposed to arginine, glycine, and methionine (Bonilla et al., 2021), because the degradation and transformation in Xuanwei ham resulted in A significant increase in the free amino acid ratio in the Xuanwei raw leg was observed, and the higher rate of transformation in Xuanwei ham resulted in a higher rate of decrease in the creatine content of Xuanwei ham than that of Jinhua ham (Zhou et al., 2021). Taurine, a non-protein amino acid, was found to be 1.43 times more abundant in the Xuanwei raw leg than in the Xuanwei ham, and 1.61 times more abundant in the Xuanwei raw leg than in the Jinhua ham. Inosine, a compound consisting of hypoxanthine and ribose (Lovászi et al., 2021), exhibited a decline in content concomitant with an increase in hypoxanthine content from raw leg to ham. This phenomenon can be attributed to the degradation of inosine, a process that yields hypoxanthine (Wang et al., 2020; Zhang, Cao, et al., 2025). The notable difference in taurine retention rates between XW and JH hams prompts the question of its cause. While the primary driver is undoubtedly the vastly higher initial content in XW raw legs, the processing conditions, particularly the salting stage, likely modulate the extent of its retention. Taurine is a highly water-soluble molecule. The more intensive salting process of JH ham—characterized by higher salt content (8–10 % vs. 6–7 %) and a longer duration (25–35 days vs. 15–20 days)—would be expected to induce more profound cellular dehydration and consequently a greater efflux of water-soluble compounds, including taurine, into the exuded brine. This could contribute to the lower absolute retention of taurine observed in the final JH product. Therefore, the salting parameters are a plausible contributing factor to the differential retention of water-soluble metabolites like taurine, alongside the fundamental breed-specific differences in initial composition (Cônsolo et al., 2020).

### 3.4 Differences in the composition of small molecular metabolites between Xuanwei ham and Jinhua ham

**Fig. 2:**
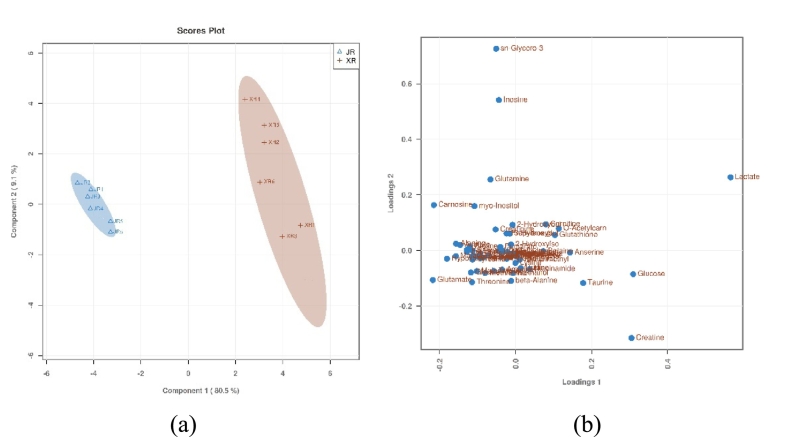
PLS-DA score map (a) and load map (b) of small molecular metabolites in the raw meat of Xuanwei ham and Jinhua ham.

As illustrated by the PLS-DA score plot (Fig. 2a), the two sample groups are evidently separated, with the PLS-DA loading plot (Fig. 2b) demonstrating the contribution of metabolites to the differences between the sample groups. It is evident that the greater the contribution of metabolites to sample group differentiation, the more distant they are from the centre point. The PLS-DA loading plot has been shown to be a valuable tool for identifying significant metabolites that contribute to the distinction between sample groups, as evidenced by the findings of an earlier investigation (Tian et al., 2022).

**Fig. 3:**
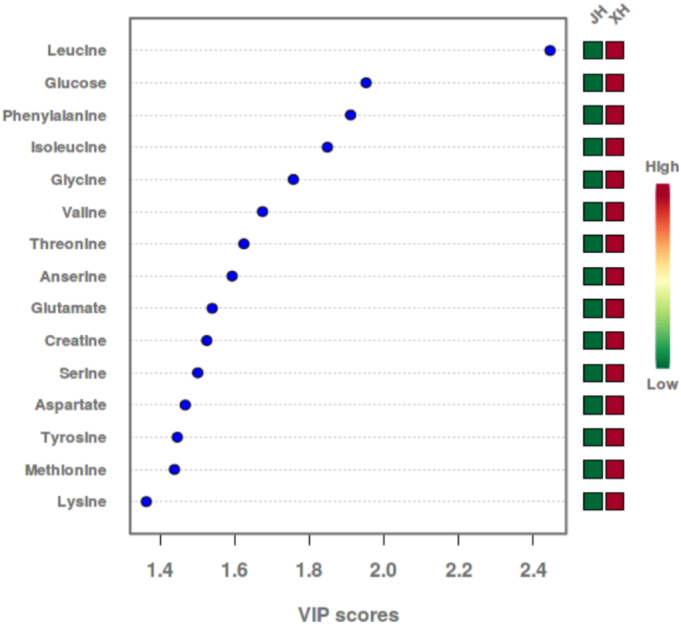
VIP diagram of PLS-DA analysis of small molecular metabolites in Xuanwei ham and Jinhua ham.

The PLS-DA VIP (Variable Importance in Projection) plot is employed to illustrate the significance of variables and their function in sample differentiation (see Fig. 3). The PLS-DA model revealed that Xuanwei ham was enriched with a suite of metabolites including leucine, glucose, glutamate, aspartate, and lysine. From a flavor chemistry perspective, this specific combination provides a compelling mechanistic explanation for its ‘light and delicate’ sensory profile:Umami Synergy: Glutamate and aspartate are well-established umami compounds. Their presence, along with lysine (which can enhance savory notes), likely creates a strong and synergistic umami foundation, contributing to the ‘delicate’ and savory taste.Sweetness and Complexity: Glucose provides a mild, background sweetness that can round out the flavor profile and balance saltiness. Valine, threonine, and serine also impart subtle sweet notes.Flavor Modulation and Balance: Taurine is particularly noteworthy. While not intensely flavorful itself, it is a renowned umami enhancer and taste modulator, effectively reducing the perception of bitterness and saltiness while promoting a smoother, more continuous mouthfeel. This is highly consistent with the ‘light’ and well-balanced description.

**Fig. 4:**
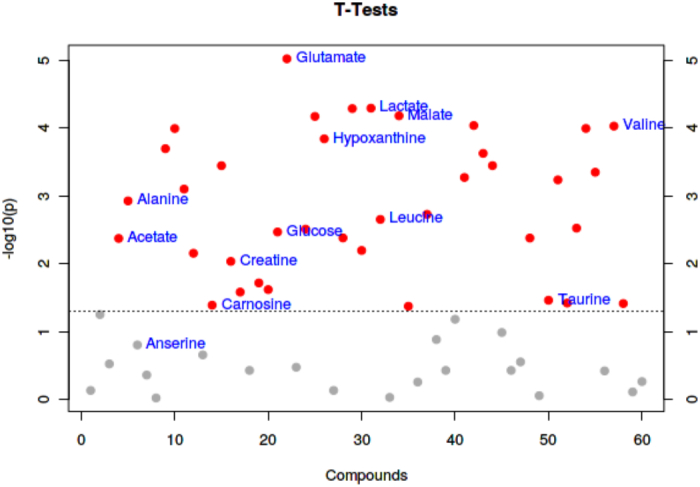
*T*-TEST diagram of PLS-DA analysis of small molecular metabolites in the raw meat of Xuanwei ham and Jinhua ham.

**Fig. 5:**
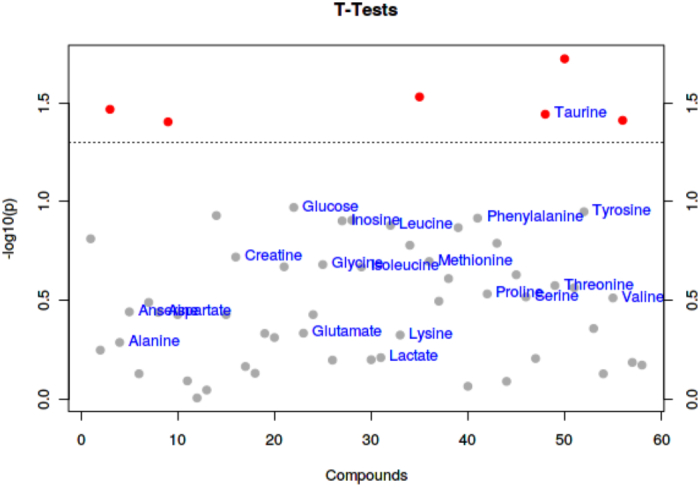
T-TEST diagram of PLS-DA analysis of small molecular metabolites in Xuanwei ham and Jinhua ham.

While this study did not include sensory evaluation experiments (e.g., descriptive analysis, electronic tongue) or taste threshold determinations to directly validate the individual contribution of each compound, the observed metabolic signature aligns perfectly with the known functionalities reported extensively in the literature(Cai et al., 2023,Li, Zheng, et al., 2024).The accumulation of these taste-active compounds in Xuanwei ham provides a strong, albeit indirect, chemical basis for its characteristic flavor.Future work employing targeted quantitation of these key metabolites, combined with sensory analysis (e.g., electronic tongue, descriptive panel) and calculation of Taste Activity Values (TAVs), is essential to conclusively establish dose-response relationships and causal contributions to the overall flavor perception.

as seen in Fig. 4: glutamate, lactate, hypoxanthine, valine, malic acid, alanine, acetic acid, leucine, glucose, creatine, carnosine, and taurine. As demonstrated in Fig. 5, taurine was the metabolite that varied significantly between the ham groups (Qaradakhi et al., 2020). Taurine is a non-protein amino acid that lowers blood cholesterol levels and treats and prevents hypercholesterolemia.

While this study has delineated the comprehensive compositional differences between Xuanwei and Jinhua hams, the critical question remains: which of these differences constitute the core metabolic basis for their distinct flavor profiles?Based on our results, we propose a mechanistic framework driven by two divergent metabolic pathways:The significantly higher levels of taurine (1.77-fold), glutamate, and lysine in XW ham are pivotal. Taurine is a well-documented umami enhancer and taste modulator. Its synergy with glutamate (also elevated in XW) likely forms the biochemical foundation for the characteristic ‘light, delicate, and lingering umami’ sensation of XW ham, while also mitigating salty and bitter notes. The drastically reduced PUFA/SFA ratio in JH ham signals intense lipid oxidation. This, coupled with its higher-temperature processing, promotes the generation of a vast array of volatile carbonyl compounds (aldehydes, ketones) from FFA breakdown and through Maillard reactions involving amino acids and reducing sugars. This pathway is the primary driver for the ‘strong and robust’ aroma profile characteristic of JH ham.

Thus, the flavor differentiation is not attributable to a single metabolite, but to the dominance of these two distinct metabolic routes: one favoring non-volatile taste-active compounds (XW), and the other favoring volatile aroma compounds (JH).

### 3.5 Correlations between environmental parameters and metabolic shifts

The divergent temperature and humidity profiles between Xuanwei (XW) and Jinhua (JH) regions (Fig. 1) are not merely background conditions but rather the main driving factor of the metabolic outcome differences between the two. To solidify the ‘environment-metabolism-flavor’ logic, we infer strong correlations based on our data and established biochemical principles: The drastic decline in the PUFA/SFA ratio in JH ham (Table 3) and its significantly lower content of oxidation-sensitive PUFAs like linoleic acid (C18:2n6c) and arachidonic acid (C20:4n6)(Table 2)exhibit a direct correlation with its exposure to higher temperatures, particularly during the critical ∼30 °C fermentation stage. Lipid oxidation kinetics are well-known to follow an exponential relationship with temperature (Q10 effect). The higher temperature in JH provides the activation energy for the rapid oxidation of these unsaturated fatty acids, leading to their depletion and the generation of volatile aldehydes and ketones that define its ‘strong aroma’. Conversely, the cooler environment of XW acts as a brake on these reactions, preserving a higher proportion of PUFAs and contributing to its ‘lighter’ lipid-derived flavor profile.

The consistently higher humidity in JH, coupled with its high-temperature stage, creates an ideal environment for the proliferation of mold and bacteria. Microbial communities are a rich source of exoproteases and peptidases. The significantly higher levels of free amino acids (e.g., glutamate, lysine) in JH ham (Table 5) are a direct metabolic consequence of this enhanced microbial proteolytic activity. The moisture facilitates the diffusion of these enzymes and their substrates, intensifying proteolysis.The retention of highly water-soluble metabolites like taurine is inversely related to the intensity of the salting and drying process. The higher temperatures and longer salting duration in JH likely promote greater moisture loss and efflux of water-soluble compounds, contributing to its lower final taurine content compared to XW. The milder conditions in XW better preserve these compounds.

In conclusion, the specific metabolic fingerprints of XW and JH hams—characterized by differential preservation of PUFAs and accumulation of free amino acids—are not random but are directly interpretable through the lens of their distinct environmental processing parameters, which modulate the rates of key biochemical reactions like lipid oxidation and proteolysis.

## 4 Conclusion

This study demonstrates that the distinct flavor profiles of Xuanwei (XW) and Jinhua (JH) hams arise from fundamental differences in lipid oxidation and metabolite composition. XW ham exhibits greater oxidative stability, retaining significantly more polyunsaturated fatty acids—particularly linoleic acid—along with higher levels of taste-active metabolites such as taurine, glutamate, and lysine, which contribute to its mild and umami-rich flavor. In contrast, JH ham underwent substantial lipid degradation during its high-temperature fermentation process, leading to a sharp decline in PUFA content and promoting the formation of volatile aroma compounds. These metabolic differences, driven by raw material and processing conditions, provide a biochemical basis for their characteristic flavors and support targeted improvements in traditional dry-cured ham production. The findings of this study provide a theoretical foundation for comparing the flavors of Xuanwei and Jinhua hams. To directly validate the proposed flavor mechanisms, future work will employ targeted quantitation of the key taste-active compounds (e.g., taurine, glutamate) and aroma compounds (e.g., hexanal, nonanal) identified in this study, coupled with sophisticated sensory evaluation techniques (e.g., descriptive analysis, electronic nose, and tongue) and omission tests to establish definitive causal relationships.

## CRediT authorship contribution statement

**Ruwei Ren:** Writing – original draft, Supervision, Methodology, Conceptualization. **Ling Li:** Software, Data curation, Conceptualization. **Guiying Wang:** Writing – review & editing, Visualization, Validation. **Jia Liu:** Formal analysis, Data curation. **Nannan Zhou:** Investigation, Formal analysis. **Wen Xun:** Software, Methodology. **Yanfei Du:** Software, Data curation. **Shuai Tang:** Software, Investigation. **Jiayan Tan:** Methodology, Data curation. **Guozhou Liao:** Supervision, Project administration, Funding acquisition.

## Declaration of competing interest

The authors declare that they have no known competing financial interests or personal relationships that could have appeared to influence the work reported in this paper.

## Data Availability

Data will be made available on request.

## Appendix A Supplementary data

Supplementary material Image 1
