# Supplementary material for: 1H NMR-based small molecular metabolites characterization and free fatty acid composition of Xuanwei ham and Jinhua ham
Source: Food Chem X. 2025 Sep 23;31:103071. doi: 10.1016/j.fochx.2025.103071 (PMC12509130; doi:10.1016/j.fochx.2025.103071)
Supplement: Supplementary file 1 — Supplementary material [file mmc1.docx]

Table S1 Metabolites identified and analyzed in raw meat and the number of ^1^H NMR spectra

| No | Metabolite | No | Metabolite |
| --- | --- | --- | --- |
| 1 | 2-Hydroxybrate | 31 | Lactate |
| 2 | 2-Hydroxyisovalerate | 32 | Leucine |
| 3 | 3-Hydroxybutyrate | 33 | Lysine |
| 4 | Acetate | 34 | Malate |
| 5 | Alanine | 35 | Malonate |
| 6 | Anserine | 36 | Methano |
| 7 | Arginine | 37 | Methionine |
| 8 | Asparagine | 38 | N, N-Dimethylglycine |
| 9 | Aspartate | 39 | N-Methylhydantoin |
| 10 | Betaine | 40 | Niacinamide |
| 11 | Butyrate | 41 | Nicotinate |
| 12 | Cadaverine | 42 | O-Acetylcarnitine |
| 13 | Carnitine | 43 | Phenylalanine |
| 14 | Carnosine | 44 | Proline |
| 15 | Choline | 45 | Propylene glycol |
| 16 | Creatine | 46 | Pyroglutamate |
| 17 | Creatinine | 47 | Sarcosine |
| 18 | Ethanolamine | 48 | Serine |
| 19 | Formate | 49 | Succinate |
| 20 | Fumarate | 50 | Taurine |
| 21 | Glucose | 51 | Threonine |
| 22 | Glutamate | 52 | Trimethylamine |
| 23 | Glutamine | 53 | Tryptophan |
| 24 | Glutathione | 54 | Tyrosine |
| 25 | Glycine | 55 | Uracil |
| 26 | Hypoxanthine | 56 | Uridine |
| 27 | Inosine | 57 | Valine |
| 28 | Isobutyrate | 58 | Myo-Inositol |
| 29 | Isoleucine | 59 | Sn-Glycero-3-phosphocholine |
| 30 | Isopropano | 60 | Beta-Alanine |

Table S2 Metabolites identified and analyzed in ham and the number of ^1^H NMR spectra

| No | Metabolite | No | Metabolite |
| --- | --- | --- | --- |
| 1 | 3-Hydroxybutyrate | 29 | Leucine |
| 2 | Acetate | 30 | Lysine |
| 3 | Alanine | 31 | Malonate |
| 4 | Anserine | 32 | Methano |
| 5 | Arginine | 33 | Methionine |
| 6 | Asparagine | 34 | N,N-Dimethylglycine |
| 7 | Aspartate | 35 | N-Methylhydantoin |
| 8 | Betaine | 36 | Niacinamide |
| 9 | Butyrate | 37 | Nicotinate |
| 10 | Cadaverine | 38 | Phenylalanine |
| 11 | Carnitine | 39 | Proline |
| 12 | Carnosine | 40 | Propylene glycol |
| 13 | Choline | 41 | Pyroglutamate |
| 14 | Creatine | 42 | Sarcosine |
| 15 | Creatinine | 43 | Serine |
| 16 | Uridine | 44 | Succinate |
| 17 | Ethanolamine | 45 | Taurine |
| 18 | Formate | 46 | Threonine |
| 19 | Fumarate | 47 | Trimethylamine |
| 20 | Glucose | 48 | Tryptophan |
| 21 | Glutamate | 49 | Tyrosine |
| 22 | Glycine | 50 | Uracil |
| 23 | Hypoxanthine | 51 | Uridine |
| 24 | Inosine | 52 | Valine |
| 25 | Isobutyrate | 53 | Myo-Inositol |
| 26 | Isoleucine | 54 | Sn-Glycero-3-phosphocholine |
| 27 | Isopropano | 55 | Beta-Alanine |
| 28 | Lactate |  |  |
